# Lungworm Infections in German Dairy Cattle Herds — Seroprevalence and GIS-Supported Risk Factor Analysis

**DOI:** 10.1371/journal.pone.0074429

**Published:** 2013-09-05

**Authors:** Anne-Marie Schunn, Franz J. Conraths, Christoph Staubach, Andreas Fröhlich, Andrew Forbes, Thomas Schnieder, Christina Strube

**Affiliations:** 1 Institute for Parasitology, University of Veterinary Medicine Hannover, Hannover, Germany; 2 Friedrich-Loeffler-Institut, Federal Research Institute for Animal Health, Institute of Epidemiology, Wusterhausen, Germany; 3 Merial, Lyon, France; Auburn University, United States of America

## Abstract

In November 2008, a total of 19,910 bulk tank milk (BTM) samples were obtained from dairy farms from all over Germany, corresponding to about 20% of all German dairy herds, and analysed for antibodies against the bovine lungworm *Dictyocaulus viviparus* by use of the recombinant MSP-ELISA. A total number of 3,397 (17.1%; n = 19,910) BTM samples tested seropositive. The prevalences in individual German federal states varied between 0.0% and 31.2% positive herds. A geospatial map was drawn to show the distribution of seropositive and seronegative herds per postal code area. ELISA results were further analysed for associations with land-use and climate data. Bivariate statistical analysis was used to identify potential spatial risk factors for dictyocaulosis. Statistically significant positive associations were found between lungworm seropositive herds and the proportion of water bodies and grassed area per postal code area. Variables that showed a statistically significant association with a positive BTM test were included in a logistic regression model, which was further refined by controlled stepwise selection of variables. The low Pseudo R^2^ values (0.08 for the full model and 0.06 for the final model) and further evaluation of the model by ROC analysis indicate that additional, unrecorded factors (e.g. management factors) or random effects may substantially contribute to lungworm infections in dairy cows. Veterinarians should include lungworms in the differential diagnosis of respiratory disease in dairy cattle, particularly those at pasture. Monitoring of herds through BTM screening for antibodies can help farmers and veterinarians plan and implement appropriate control measures.

## Introduction

The disease dictyocaulosis in cattle, also known as parasitic bronchitis or “husk”, is caused by the lungworm *Dictyocaulus viviparus*. This parasitosis is nowadays recognized as a problem in both, calves and adult cattle in temperate regions throughout the world. In the last century, dictyocaulosis was commonly considered as a disease of pastured, first-year grazing calves. However, in the 1990s an increase in the incidence of disease was recorded [1], [2] and coincidently there was a substantial increase in the proportion of second year grazing calves or adult cows [1], [3]. Both dairy and beef cattle can be affected [4], [5]. Outbreaks in dairy cattle herds can cause considerable economic losses due to reduced milk production, body weight and fertility or even death of infected animals on the one hand and expenses for laboratory diagnosis and treatments on the other hand [6]–[8]. Total costs for lungworm outbreaks in dairy herds were estimated by Holzhauer et al. [8] at €159 and €167 per cow, whereas Woolley et al. [6] calculated the potential economic cost of a moderate outbreak, affecting the whole herd, at roughly 300 € per cow. To gain information about the distribution of lungworm infections in dairy cattle, several seroepidemiological studies have been carried out to assess the epidemiological situation regarding the parasite in various countries. In The Netherlands, 50% of pastured dairy herds in 1981 and 85% in 1982 were found to be positive for lungworm antibodies in serum samples, while values were 14% and 57%, respectively, for zero-grazing farms [9]. Significantly lower prevalences were reported more recently from other European countries: in the northern German region East Frisia 21.1% of dairy herds tested had positive antibodies against *D. viviparus* in bulk tank milk (BTM) samples on at least one sampling occasion in 2008 [10]. A study conducted 2006 in Flanders, Belgium, reported a similar percentage of 19.6% of BTM-seropositive dairy herds [11] and for Sweden in 2008 it was shown that 18% of organic dairy herds and 9% of conventional dairy herds were BTM-seropositive [12].

The aim of the present survey was to determine the prevalence of *D. viviparus* infections in dairy cattle herds in Germany and, for the first time, to evaluate potential spatial risk factors that may influence the prevalence of lungworm infections. Therefore, about 20,000 BTM samples from regions all over Germany were tested with the MSP-ELISA, which can be used for diagnosing antibodies against the bovine lungworm in serum as well as individual milk and BTM samples [13], [14]. A recently field study in 15 dairy herds in Germany allowed the cut-off for lungwoem positivity to be adjusted and showed that this ELISA is a useful method for testing BTM samples in epidemiological surveys [15].

## Materials and Methods

### Study Area and Dairy Cattle Farming

Germany is located in Central Europe and with a surface area of 357,123 km^2^ it is the sixth-largest country in Europe. About one third of the land is covered by forest and woodlands and about 50% of the land is used for agricultural production [16] Dairy cattle farming represents an important pillar of German agriculture, and in the European Union, Germany is currently the largest producer of milk with a share of more than 20% [17]. The Federal Statistical Office of Germany recorded that about 4.2 million dairy cattle were kept in Germany in 2008, corresponding to 11.76 dairy cattle per km^2^ on average. Most of the German dairy farms are located in the German federal states of Bavaria and Lower Saxony. About 42% of the total stock was kept on pasture for an average time of 24 weeks per year [18].

### Study Design and Sampling Procedure

In November 2008, a total of 22,427 BTM samples were collected from all federal states of Germany (Brandenburg and the city state Berlin were handled as one federal state and samples originating from the city states Hamburg and Bremen were assigned to Lower Saxony) in cooperation with dairy factories and state dairy quality control associations (“Landeskontrollverbände”). BTM samples had been taken by these institutions for routine checks in the course of milk yield recording and aliquots were made available for the purpose of this study.

**Figure 1:**
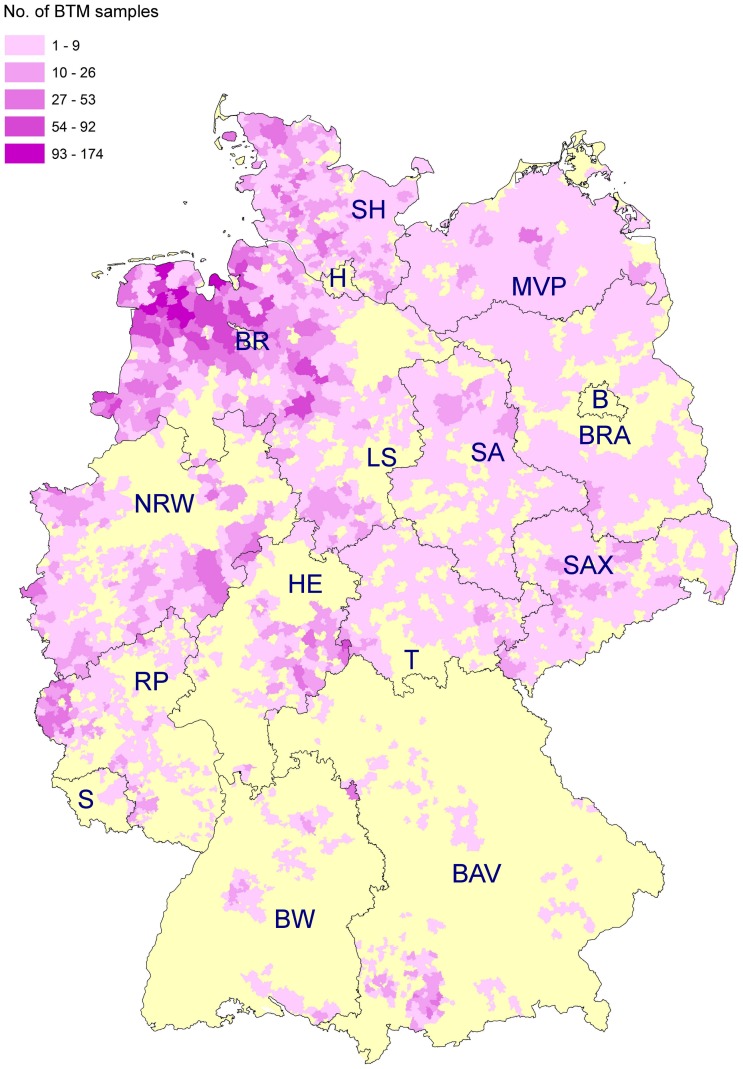
Map of Germany illustrating the number of examined BTM-samples per postal code district. The yellow colour marks postal code districts which were not sampled. Federal states are abbreviated as follows: B = Berlin, BAV = Bavaria, BR = Bremen; BRA = Brandenburg, BW = Baden-Württemberg, H = Hamburg, HE = Hesse, LS = Lower Saxony, MWP = Mecklenburg-Western Pomerania, NRW = North Rhine-Westphalia, RP = Rhineland-Palatinate, S = Saarland, SA = Saxony-Anhalt, SAX = Saxony, SH = Schleswig-Holstein, T = Thuringia.

The BTM samples were collected by the first author or send to the Institute for Parasitology of the University of Veterinary Medicine Hannover by courier. After arrival, the samples were centrifuged at 2000×*g* for 15 minutes followed by removal of the superficial fat layer and pelleted components. The remaining milk was aliquoted and stored at −20°C until tested by ELISA. Collected BTM samples were attributed to the postal code district of the respective dairy farm because the full addresses were not made available due to confidentiality issues. Samples that could not be unambiguously attributed to a postal code were excluded. Only a single BTM sample for each farm was used for further analysis. After sample preparation and data processing, a total of 19,910 BTM samples were serologically and statistically analysed. The number of samples per federal state ranged from 37 from Saarland to 8,841 from Lower Saxony. Detailed data are presented in Table 1, whereas Figure 1 shows the distribution of sampled farms (number of farms per postal code district). Altogether, 2656 postal code districts were included in the study and between 1 and 174 farms per postal code district were sampled.

**Table 1:** BTM ELISA results and dairy cattle data.

Federal state	No. of BTM samples(% of German dairyfarms in 2008)	No. of negative BTM samples	No. of positiveBTM samples	Estimated prevalence(%)	95% CI(%)	No. of dairy cows in 2008	No. of dairy farms in 2009	No. of pastured dairy herds in 2009	% pastured dairy herds in 2009
Baden-Württemberg	478 (4.1%)	462	16	3.3	2.0–5.5	360,609	11,303	4,000	35.4
Bavaria	877 (1.9%)	786	91	10.4	8.5–12.6	1,256,554	43,738	8,200	18.8
Brandenburg and Berlin	282 (33.7%)	248	34	12.1	8.6–16.6	167,110	837	300	35.8
Hesse	954 (21.4%)	875	79	8.3	6.6–10.3	151,850	4,293	2,300	53.6
Lower Saxony, Hamburg, and Bremen	8,841 (59.3%)	6,867	1,974	22.3	21.5–23.3	780,672	14,258	10,400	72.9
Mecklenburg-Western Pomerania	644 (62.5%)	550	94	14.6	12.0–17.6	174,355	999	500	50.1
North Rhine-Westphalia	2,083 (22.3%)	1,747	336	16.1	14.6–17.8	396,922	8,946	7,100	79.4
Rhineland-Palatinate	909 (33.4%)	846	63	7.0	5.4–8.8	119,150	2,629	1,700	64.7
Saarland	37 (14.5%)	37	0	0.0	0.0–11.7	14,033	251	200	79.7
Saxony	960 (57.7%)	944	16	1.7	1.0–2.8	190,781	1,616	600	37.1
Saxony-Anhalt	356 (43.7%)	245	111	31.2	26.5–36.3	128,141	756	300	39.7
Schleswig-Holstein	3,138 (56.4%)	2,585	553	17.6	16.3–19.0	373,185	5,383	4,500	83.6
Thuringia	351 (47.1%)	321	30	8.5	5.9–12.1	115,849	772	300	38.9
Total	19,910 (20.0%)	16,513	3,397	17.1	16.5–17.6	4,229,138	95,766	40,300	42.1

*D. viviparus*-antibody positive and negative BTM samples, prevalence estimates for the sampled areas in the federal states of Germany and dairy cattle data obtained from the German Federal Statistical Office. As the number of pastured dairy herds was only available for 2009, the number of dairy farms was also related to 2009.

### Bulk Tank Milk ELISA

The *D. viviparus* antibody levels in BTM samples were analyzed with a recombinant MSP-ELISA as described previously [13]. The arithmetic means of the ODs of the duplicates were converted into optical density ratios (ODRs) using the following formula:
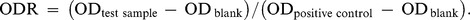



The field-adjusted cut-off value of 0.410 ODR for positivity, according to Schunn et al. [15] was chosen.

### Analysis using Geographic Information System (GIS)

Raw data were entered in Excel spreadsheets (Microsoft® Office 2003) for descriptive analysis. Spatial risk factors that might be associated with the distribution of dictyocaulosis were tested for statistically significant associations with the ELISA results. GIS analysis was carried out with the following variables:

1. Dependent variables:
  i The ODR value for each sampled farm with ODR ≥0.410 as positive (“1″) and <0.410 as negative (“0”).
2. Independent variables:
  ii The postal code of each sampled dairy farm (map scale of the postal areas 1∶250,000).
  iii Cattle and farm density as recorded in the Identification and Information System for Animals (Herkunftssicherung- und Informationssystem für Tiere, HIT, Munich, Germany).
  iv Climate raster data (1 km raster) for Germany from the German meteorological service (kindly provided by the Deutscher Wetterdienst, dwd, Offenbach, Germany): Mean daily temperature per month (°C), mean precipitation per month. The variables were grouped by quarter for 2008: quarter one (Tm1q, P1q): January to March, quarter two (Tm2q, P2q): April to June, quarter three (Tm3q, P3q): July to September, quarter four (Tm4q, P4q): October to December. In all analyses temperature was always combined with altitude, which was also used as an independent factor.
  v Land-use data (agricultural crop land, forest area [corine land cover (CLC); map scale 1∶100,000], European Environment Agency, Copenhagen, Denmark) and grassed area [CLC, ATKIS (Official Topographical Cartographic Information System, Federal Agency for Cartography and Geodesy, Frankfurt, Germany); map scale 1∶2,500].
  vi Lentic water bodies (ATKIS).

Farm and cattle density, climate and land-use data were attributed to postal code areas as the common spatial unit and maps plotted using ArcGIS software (version 9.3.1; ESRI, Redlands, CA, USA) as described [19].

### Statistical Analysis

All statistical analyses were performed using R (http://www.r-project.org/; including the libraries MASS, glmmML, lme4). A p-value of ≤0.05 was considered statistically significant.

First, all variables listed in the section above were individually tested against the ELISA results by bivariate testing. Correlation between the variables was taken into account by building models that always contained temperature and altitude, and which included a landscape categories selected on the basis of biological criteria potentially related to the life cycle of *D. viviparus.* Variables either from the CLC or ATKIS dataset in the multivariate models were used. Afterwards, a multiple logistic regression model was created. The full model contained the variables cattle density, water bodies, grassed area (ATKIS), agricultural crop land (CLC), forest area (CLC), altitude, temperature and precipitation., These components were modified by stepwise model building with automated forward and backward selection of variablesto identify the most parsimonious model. To check the model selection procedure and consistency, we also created models manually with all pairs of climate variables including altitude and landscape categories separately. Finally, a general linear mixed model with random intercept was set up on postal code level to include spatial information in the model. Pseudo-R^2^ values were calculated to assess the proportion of variability in the outcome variable (ELISA result) explained by the respective model. Receiver operating characteristics (ROC) analysis was used to further characterise the performance of the models [20].

## Results

### Prevalence of Dictyocaulosis in Germany

**Figure 2:**
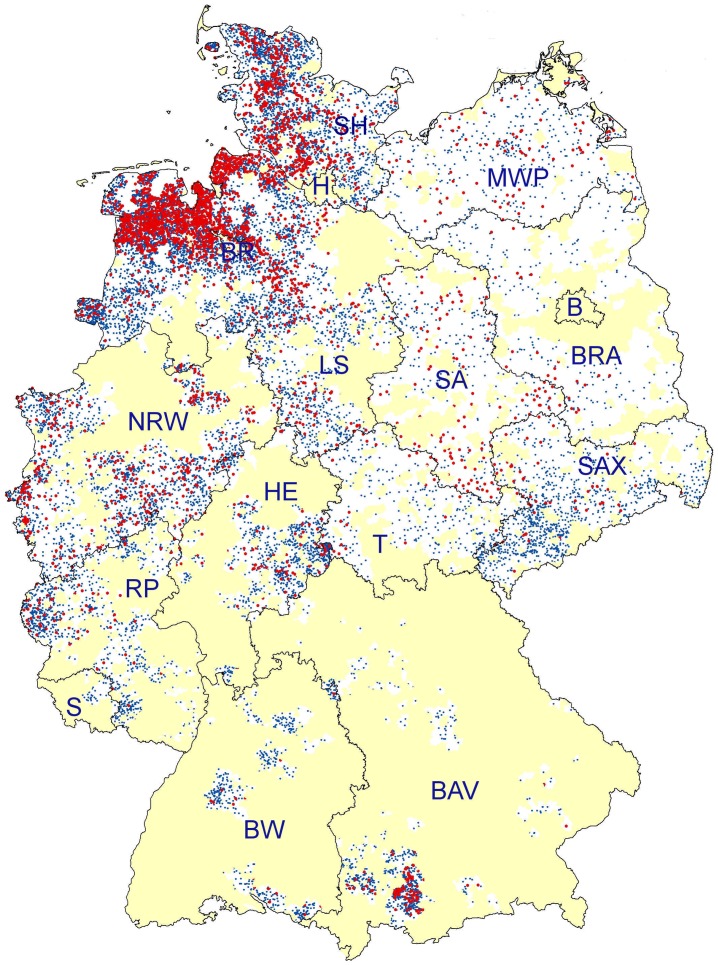
Distribution of *D. viviparus* BTM ELISA positive (red dots) and negative (blue dots) dairy herds throughout the sampled regions of Germany. Dots for BTM-positive and -negative dairy herds were randomly distributed within the respective postal code area. The white colour marks sampled postal code areas, while the yellow colour marks postal code areas which were not sampled. Federal states are abbreviated as follows: B = Berlin, BAV = Bavaria, BR = Bremen; BRA = Brandenburg, BW = Baden-Württemberg, H = Hamburg, HE = Hesse, LS = Lower Saxony, MWP = Mecklenburg-Western Pomerania, NRW = North Rhine-Westphalia, RP = Rhineland-Palatinate, S = Saarland, SA = Saxony-Anhalt, SAX = Saxony, SH = Schleswig-Holstein, T = Thuringia.

**Figure 3:**
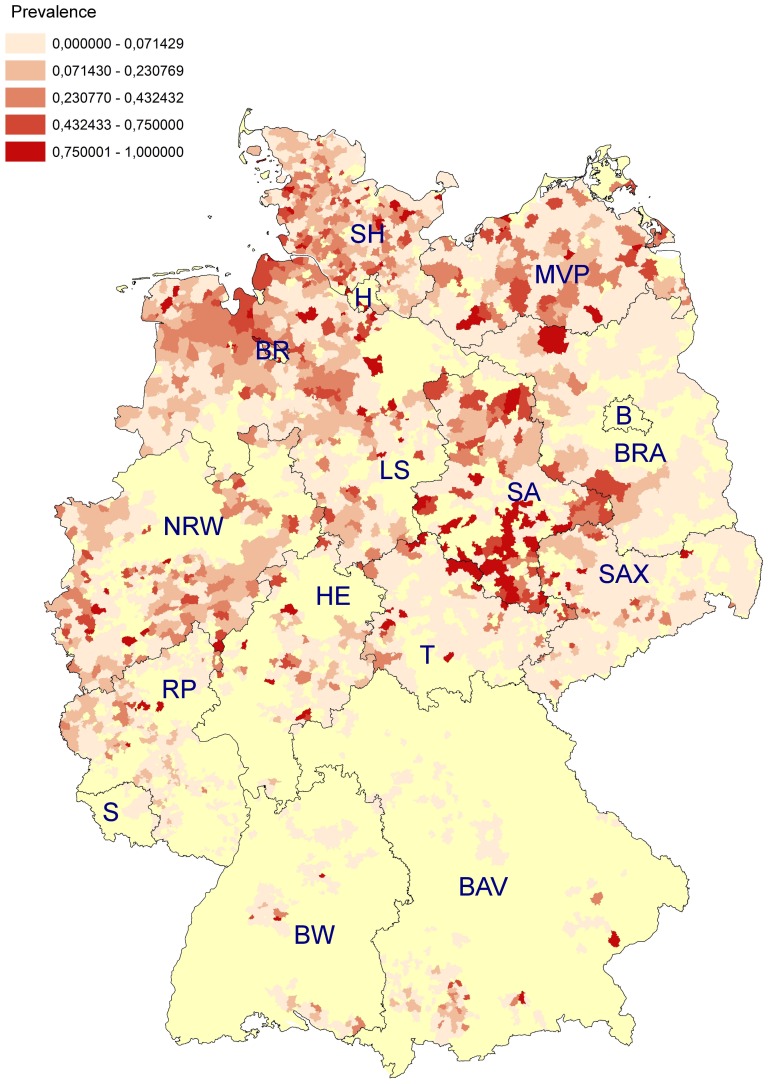
Prevalence for the several postal code districts (No. of positive samples/No. of samples per postal code district). The yellow colour marks postal code districts which were not sampled. Federal states are abbreviated as follows: B = Berlin, BAV = Bavaria, BR = Bremen; BRA = Brandenburg, BW = Baden-Württemberg, H = Hamburg, HE = Hesse, LS = Lower Saxony, MWP = Mecklenburg-Western Pomerania, NRW = North Rhine-Westphalia, RP = Rhineland-Palatinate, S = Saarland, SA = Saxony-Anhalt, SAX = Saxony, SH = Schleswig-Holstein, T = Thuringia.

A total number of 3,397 (17.1%; 95% CI: 16.5–17.6%) BTM samples tested positive for anti-*D. viviparus* antibodies. The highest percentages of seropositive dairy herds were found in Central and Northern Germany with 31.2% (95% CI: 26.5–36.3%) seropositive in the sampled areas of the federal state of Saxony-Anhalt, 22.3% (95% CI: 21.5–23.3%) in Lower Saxony and 17.6% (95% CI: 6.3–19.0%) in Schleswig-Holstein. The lowest estimated prevalences were observed in the sampled areas of Baden-Württemberg (3.3%; 95% CI: 2.0–5.5%), Saxony (1.7%; 95% CI: 1.0–2.8%), and Saarland (0.0%; 95% CI: 0.0–11.7%). Detailed results are listed in Table 1. A map of Germany illustrating the distribution of positive (red dots) and negative (blue dots) dairy herds in the analysed postal code districts is given in Figure 2, while Figure 3 shows the resulting estimated prevalences for the sampled postal code districts (no. of positive samples/no. of samples per postal code district).

### Statistical Analysis

#### (1) Bivariate comparison

The results of bivariate statistical analysis are shown in Table 2. Most of the tested variables were significantly associated with the bulk tank milk ELISA results for each farm. A positive association was observed for the variables cattle density, farm density, lentic water bodies and grassed area, whereas agricultural crop land, forest area and altitude were negatively correlated. Temperature was analysed first for each quarter individually always combined with the respective altitude and revealed a positive association for Tm1q and Tm4q, whereas Tm2q and Tm3q showed a negative association. No significant association was observed for Tm1q when each quarter was analysed individually. In the model, we further checked for an association between the results of the BTM samples and temperature during all four quarters (Tm1q – Tm4q) together, combined with the respective altitudes. In this analysis Tm1q and Tm3q revealed a positive association, whereas Tm2q and Tm4q showed a negative association. Statistically significant associations were found for the variables Tm1q and Tm2q. Concerning precipitation, a positive association was found by individual analysis for three out of four quarters, P1q, P3q and P4q whereas a negative association became evident for P2q. All precipitation quarters were significantly associated when individually analysed, whereas in the combined analysis of all quarters, only the first quarter failed to show a statistically significant association. In this analysis only P3q resulted in a positive association.

**Table 2:** Bivariate statistical analysis.

Variable		Estimate	Pr(>|z|)
Cattle density		3.245e-05	<2e-16***
Farm density		0.0027089	<2e-16***
Lentic water bodies (ATKIS)		5.46747	<2e-16***
Grassed area (ATKIS)		2.25266	<2e-16***
Grassed area (CLC)		1.69672	<2e-16***
Agricultural crop land (CLC)		−1.28341	<2e-16***
Forest area (CLC)		−2.29967	<2e-16***
Altitude		0.0022456	<2e-16***
Temperature first quarter	Tm1q	0.0075451	0.123
	Altitude	−0.0019480	<2e-16***
Temperature second quarter	Tm2q	−0.0166507	1.64e-07***
	Altitude	−0.0024112	<2e-16***
Temperature third quarter	Tm3q	−0.038995	6.36e-11***
	Altitude	−0.002748	<2e-16***
Temperature fourth quarter	Tm4q	0.027540	0.00104**
	Altitude	−0.001068	0.00461**
Temperature Tm1-4q	Tm1q	0.0846778	8.27e-11***
	Tm2q	−0.0647443	1.70e-08***
	Tm3q	0.0176859	0.2131
	Tm4q	−0.0223971	0.0689
	Altitude	−0.0003204	0.4594
Precipitation first quarter	P1q	0.008448	1.44e-13***
Precipitation second quarter	P2q	−0.018938	<2e-16***
Precipitation third quarter	P3q	0.0130757	<2e-16***
Precipitation fourth quarter	P4q	0.01285	<2e-16***

* p = ≤0.05.

** p = ≤0.01.

*** p = ≤0.001.

#### (2) Multivariate analysis

**Figure 4:**
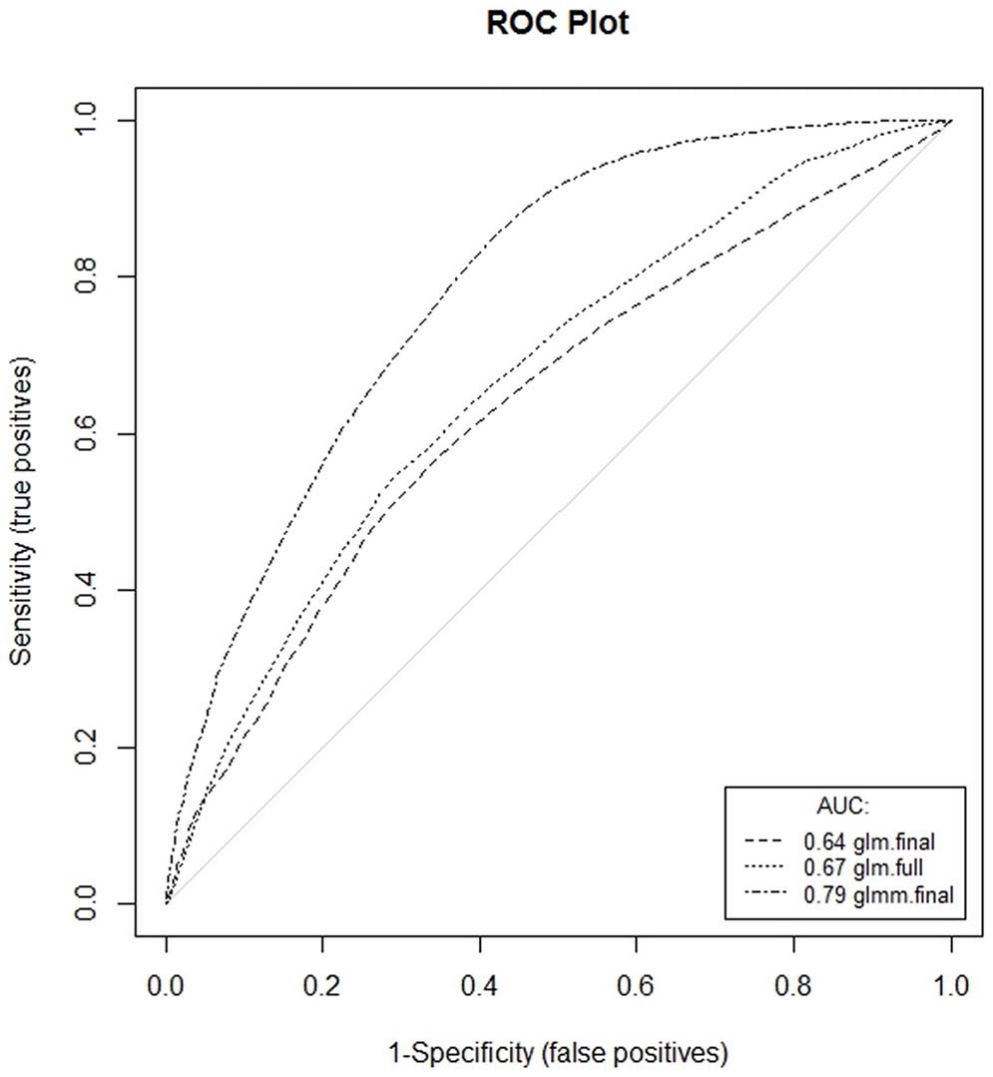
Comparison of the predictive performance of models by ROC-analysis.

Based on the results of bivariate testing, the following variables were included in the full logistic regression model: cattle density, lentic water bodies (ATKIS), grassed area (ATKIS), agricultural crop land (CLC), forest area (CLC), altitude, temperature (all quarters) and precipitation (all quarters, Table 3). The model revealed a significant association for all analysed variables except for forest area and P1q. Manually controlled stepwise selection of variables revealed that the most parsimonious model included the variables lentic water bodies and grassed area (Table 4). Evaluation of the full and final model after stepwise inclusion or exclusion of variables showed low Pseudo R^2^ values of 0.08 (Table 3) and 0.06 (Table 4) respectively, which was confirmed by empirical ROC analysis (Figure 4) of the models (AUC_full_ = 0.67 and AUC_final_ = 0.64).

**Table 3:** Multivariate statistical analysis, full logistic regression model.

Variable	Estimate	Pr(>|z|)	AIC^1^	Pseudo-R^2^
Cattle density	−7.482e-06	0.01112*	17304	0.083
Lentic water bodies (ATKIS)	1.959e+00	0.00678**		
Grassed area (ATKIS)	1.571e+00	1.44e-13***		
Agricultural crop land (CLC)	−5.680e-01	0.00147**		
Forest area (CLC)	4.840e-01	0.06092		
Altitude	−3.786e-03	5.24e-11***		
Tm1q	6.978e-02	1.97e-06***		
Tm2q	−8.255e-02	7.99e-10***		
Tm3q	9.629e-02	3.97e-06***		
Tm4q	−8.240e-02	2.44e-07***		
P1q	4.709e-04	0.83123		
P2q	1.562e-02	3.28e-07***		
P3q	1.063e-02	1.26e-09***		
P4q	−9.069e-03	6.39e-05***		

* p = ≤0.05.

** p = ≤0.01.

*** p = ≤0.001.

1 Akaike Information Criterion.

**Table 4:** Multivariate statistical analysis, final logistic regression model after controlled stepwise selection of variables.

Variable	Estimate	Pr(>|z|)	Null deviance	Residual deviance	AIC^1^	Pseudo-R^2^
Lentic water bodies (ATKIS)	3.57652	1.46e-07***	18289	17597	17603	0.057
Grassed area (ATKIS)	2.19072	<2e-16***				

* p = ≤0.05.

** p = ≤0.01.

*** p = ≤0.001.

1 Akaike Information Criterion.

#### (3) General linear mixed model with random intercept

Based on the assumption that farms in the same postal code area have a similar environment and the same climatic conditions, a model was constructed to minimize the effects of non-independent variables. Modelling included a random intercept on the postal code level and the variables water bodies and grassed areas. The model confirmed the impact of the variables lentic water bodies and grassed area and gained additional value through inclusion of a random intercept for spatial information (Table 5) as illustrated also by the increase of the AUC value to 0.79 (Figure 4).

**Table 5:** General linear mixed model with random intercept.

Variable	Estimate	Pr(>|z|)	Residual deviance	AIC^1^
Lentic water bodies (ATKIS)	4.26463	9.46e-05***	17164	17172
Grassed area (ATKIS)	2.31100	<2e-16***		
Random intercept (Variance = 0.66029; SD = 0.81258)		<2e-16***		

* p = ≤0.05.

** p = ≤0.01.

*** p = ≤0.001.

1 Akaike Information Criterion.

## Discussion

Infections with the cattle lungworm are common in cattle on pasture throughout the temperate regions of Europe [4], [9]–[12], [21]–[23]. The present study is the first to assess the distribution of dictyocaulosis in dairy farms all over Germany and, at the same time, the first providing information on geographical and epidemiological risk factors for the parasitosis. BTM samples of about 20,000 dairy herds were analysed using the recombinant MSP-ELISA [13], [14]. When applied to BTM, this ELISA ensures the detection of herds with an in-herd prevalence of ≥20% with a sensitivity of 100% and a specificity of 97.3%, [15]. As sample collection for the present study depended on the cooperation of dairy factories and state dairy quality control associations, the selection of dairy farms was not completely random, but may have been influenced by the objectives of milk quality control. It is unlikely, however, that the selection process biased the sampling with respect to the prevalence of *D. viviparus* infections. It can therefore be assumed that the data obtained yield reasonably reliable prevalence estimates. The overall estimated prevalence of *D. viviparus* in dairy herds in Germany was 17.0%. This is comparable to findings by Bennema et al. [11] who reported 19.6% of Flemish dairy herds, which were also sampled in autumn, to be positive for *D. viviparus* BTM antibodies. Within individual German federal states, prevalences ranged between 0.0% and 31.2%. As expected, high lungworm incidences in individual federal states were often associated with a high percentage of dairy herds having access to pasture. So Lower Saxony, where about 73% of dairy herds are pastured, showed the second highest lungworm prevalence in the study (22.3%). Likewise, high seroprevalence rates were found in the federal states Schleswig-Holstein (17.6% positives) and North Rhine-Westphalia (16.1% positives), where about 84% and 80% dairy herds have access to pasture. However, in Saarland and Rhineland-Palatinate the estimated prevalence was 0.0% and 7.0%, respectively, although about 80% and 65% of herds are pastured. Interestingly, the highest prevalence was found in the federal state Saxony-Anhalt, where only about 40% of the dairy herds have access to pasture. When comparing the estimated seroprevalences for federal states it has to be taken into account that BTM samples provided by the dairy factories and state dairy quality control associations represented the entire population of their customers (i.e. the dairy farms), which included an unknown proportion of samples from zero-grazing farms. Consequently, the seroprevalence in pastured dairy herds might be considerably higher in individual federal states, particularly when the number of available BTM samples and geographic coverage was low in this study. The prevalence in the southern federal states of Bavaria and Baden-Württemberg, where only 1.9% and 4.1% of the dairy herds were sampled, should be interpreted with caution. By contrast, a high percentage of the dairy herds were sampled and a high geographic coverage was achieved in the northern and eastern federal states. Furthermore, regional differences should not be underestimated when referring to prevalence data at the level of the federal states. In a seasonal study conducted in the East Frisia, a region located in Lower Saxony, only 6.6% lungworm positive BTM samples were observed in November 2008 [10]. Even after Rogan-Gladen correction of the prevalence estimate, only 7.4% of dairy herds were assigned as seropositive [15], whereas for the whole of Lower Saxony a three times higher prevalence (22.3%) was observed in the present study.

Potential risk factors contributing to infections with the bovine lungworm were statistically analysed. Lungworm risk factors, i.e. those that were statistically significantly associated with a positive ELISA result and had coefficients >2, were only found for the variables lentic water bodies and grassed area. This was confirmed by the stepwise model building process and in the model with random intercept, which also included spatial information (post code areas). The variable “grassed area”, which as grassland represents potential cattle pastures, mirrors the pasture-born character of the parasitosis, whereas the presence of lentic water bodies may influence the survival of larvae on pasture or it could be a surrogate for topographical features that favour pasture-based dairy production. Free-living lungworm larvae are strongly dependent on humidity insofar as at temperatures above 8°C larvae do not survive 3 hours of dehydration [24]. Permanent soil moisture and thus lungworm larvae survival may be enhanced by the proximity of water bodies, whereas rainfall, as an inconsistent event and does not guarantee permanent moisture. Together with the relatively low spatial variation of precipitation in Germany, this might have contributed to the fact that the variable “precipitation” is not included in the final model. Moreover, in both the bivariate and multivariate logistic regression (cf. Table 2 and 3), the values of the parameter estimates are small, indicating a very limited impact of the factors on the infection risk, despite statistical significance, which is a result of the large sample size. As in lungworms, risk factor assessment for liver fluke infections in Germany also identified grassed area and water bodies, but not rainfall, as positively associated with fasciolosis [19]. However, in neighbouring Belgium rainfall was identified as a positive predictor for *F. hepatica* infections [25], and McCann et al. [26], [27] reported the same for England and Wales. Kuerpick et al. [19] highlighted the importance of developing individual models for different countries or regions because such models may not be simply interchangeable within the same climatic zone. It is therefore possible that lungworm risk factor analyses in other temperate European countries might find rainfall as a significant predictor for dictyocaulosis. The positive association between dictyocaulosis and lentic water bodies might have contributed to regional variations in prevalence, even within rather small geographical areas as observed for the region East Frisia vs. whole Lower Saxony. However, this variable is one among many influencing dictyocaulosis in dairy cows. In this context it has to considered that the final model had a Pseudo-R^2^ of only 0.06 and the model including all variables a Pseudo-R^2^ of 0.08. We also performed risk factor analysis based on the obsolete cut-off of 0.493 ODR suggested by Fiedor et al. [13], but the resulting data (not shown) revealed only minor differences to the outcome reported here. The low Pseudo-R^2^ values indicate that the occurrence of lungworm positive ELISA results is substantially influenced by random effects or additional, unrecorded factors, e.g. management-associated factors such as pasture management and anthelmintic treatment strategies.

## Conclusions

The results of the present survey demonstrate that bovine lungworm infections of adult dairy cattle are common in Germany. Indeed, dictyocaulosis in dairy herds is more frequent than expected from previous prevalence data obtained for the month of November in East Frisia, a German region with a high density of pastured dairy cattle [10]. Statistically significant associations were obvious between seropositivity to *D. viviparus* in BTM samples and the proportion of grassed area and lentic water bodies and, so these variables can be considered as potential risk factors for dictyocaulosis. Increased attention should be paid to lungworm infections in adult cows and veterinarians should consider this parasitosis in the event of respiratory symptoms in dairy herds. Suspected lungworm infections can be substantiated by fecal examination or ELISA testing of clinically affected individuals. It has to be kept in mind, however, that the presence of antibodies does not necessarily indicate an existing infection as antibodies against lungworms may persist for up to 6 months post-exposure [13], [14], [28]. On the other hand, the presence of antibodies may reveal subclinical lungworm infections within a herd [29], [30], which may subsequently evolve into clinical dictyocaulosis. As pasturing of cattle has a positive impact on animal welfare and, at the same time, is an increasing demand of customers, one needs to accept the presence of this pasture-borne parasitosis. Farmers and veterinarians should consider monitoring dairy herds by BTM ELISA screening at regular intervals. When results of this test are positive, control measures such as treatment at the latest by the time of removal from pastures should be implemented to prevent year-to-year transmission of lungworm infections [31], which are mainly maintained by carrier animals instead of larvae overwintering on pasture.
